# Experiments Suggest that Simulations May Overestimate Electrostatic Contributions to the Mechanical Stability of a Fibronectin Type III Domain

**DOI:** 10.1016/j.jmb.2007.06.015

**Published:** 2007-08-24

**Authors:** Sean P. Ng, Jane Clarke

**Affiliations:** Cambridge University Department of Chemistry, MRC Centre for Protein Engineering, Lensfield Rd, Cambridge, CB2 1EW, UK

**Keywords:** AFM, atomic force microscopy, SMD, steered molecular dynamics, fnIII, fibronectin type III, FNfn10, the tenth fnIII domain of human fibronectin, TNfn3, the third fnIII domain of human tenascin, AFM, MD simulations, titin, forced unfolding, extracellular matrix

## Abstract

Steered molecular dynamics simulations have previously been used to investigate the mechanical properties of the extracellular matrix protein fibronectin. The simulations suggest that the mechanical stability of the tenth type III domain from fibronectin (FNfn10) is largely determined by a number of critical hydrogen bonds in the peripheral strands. Interestingly, the simulations predict that lowering the pH from 7 to ∼4.7 will increase the mechanical stability of FNfn10 significantly (by ∼33 %) due to the protonation of a few key acidic residues in the A and B strands. To test this simulation prediction, we used single-molecule atomic force microscopy (AFM) to investigate the mechanical stability of FNfn10 at neutral pH and at lower pH where these key residues have been shown to be protonated. Our AFM experimental results show no difference in the mechanical stability of FNfn10 at these different pH values. These results suggest that some simulations may overestimate the role played by electrostatic interactions in determining the mechanical stability of proteins.

Fibronectin is an extracellular matrix protein composed of three types of repeating domains (type I, type II and type III). The type III (fnIII) domains (which are ubiquitous in many multidomain mechanical proteins) in particular have been found to play a pivotal role in regulating and mediating physiological functions of cells. This is achieved through the interaction of various domains with the integrin family of cell surface receptors.^1^ One of the critical interactions in fibronectin is the binding of the RGD (Arg-Gly-Asp) motif in the tenth fnIII domain of fibronectin (FNfn10) to cell-surface integrins. A linear RGD-peptide has been shown to bind (with reduced binding strength and selectivity) to different members of the integrin family.^2^ It may be the dynamic structure of these domains that determines the functional states of the protein. Indeed, it has been suggested that force-induced conformational change of these fnIII domains may be crucial in transmitting cellular signals.^3^ Thus knowledge of how fnIII domains respond to mechanical forces and their mechanical resistance to conformational change is of importance to understand the function of fibronectin at the molecular level.

A number of studies have investigated the mechanical properties of the fnIII domains of fibronectin by both experiment^4,5^ and simulation.^6–8^ Using steered molecular dynamics (SMD) simulations, Vogel and co-workers^6^ predicted a “mechanical hierarchy” of a number of fnIII domains that is in reasonable qualitative agreement with the hierarchy obtained from the atomic force microscopy (AFM) forced unfolding experiments.^4^ One of the most mechanically weak fnIII domains is the tenth fnIII domain of human fibronectin, FNfn10, although, interestingly, this is thermodynamically the most stable fnIII domain to have been studied to date.^9^^,^^10^ The simulations suggested that the mechanical stability of FNfn10 is largely determined by the hydrogen bonds between the A and B strands around Arg6 and Asp23 (Figure 1). Recent solution studies by Koide and co-workers^10^ showed that FNfn10 becomes more thermodynamically stable at lower pH (<5.0) as a consequence of the protonation of three negatively charged residues: Asp7, Asp23 and Glu9, which have raised p*K*_a_ values of 5.54, 5.40 and 5.25, respectively; they are essentially fully protonated below pH 4.7. Using their simulations, Vogel and co-workers^6^ showed that protonation of these side-chains allows them to move closer together to form side-chain–side-chain hydrogen bonds. They suggested that this stabilizes interactions between the A and B strands, resulting in a significant increase in the unfolding force in simulations at pH 4.7 even though it is well established that there is no correlation between mechanical stability and thermodynamic stability.^11^^,^^12^ This interesting result suggests that the mechanical stability of fibronectin might be modulated by a change in the pH of the tissues.

We tested this prediction by monitoring the unfolding force of FNfn10 at different pH values: pH 4.5 (50 mM acetate), pH 5.0 (50 mM acetate) and pH 7.0 (50 mM phosphate). It is important to note that Asp7, Asp23 and Glu9 are fully protonated at pH 4.5 as shown by NMR experiments carried out by Koide and co-workers.^10^ Any increase in mechanical stability due to the protonation of these three residues, as shown in the SMD simulation, should be observed in the AFM experiments. Forced unfolding experiments were performed for a polyprotein containing eight repeats of the FNfn10 domain.

Figure 2 shows a “typical” force-extension trace. As has previously been observed^5^ Fnfn10 unfolds either by a two-state unfolding mechanism (N → U, where N is the native state and U the unfolded state) or a three-state unfolding mechanism *via* an intermediate (I) (N → I → U). The unfolding forces for three transitions could thus be determined: *F*_N→U_, the force of unfolding of N directly to U; *F*_N→I_, the force of unfolding of N to I; and *F*_I→U_, the force of unfolding of I to U (Figure 2).

The intermediate, I, has been shown, using site-directed mutagenesis, to be a species with the A-strand detached.^5^ Thus one might expect, if the simulations of the unfolding of FNfn10 are correct, that both the N → U and N → I transitions will be at higher force at lower pH due to the new stabilising interactions between the A and B strands. However, since the A strand is detached in I, any stabilizing interactions between the A and B strands should not affect the mechanical stability of the intermediate, and *F*_I→U_ is expected to be unaffected by pH. Furthermore, the unfolding forces of N → U are higher than those of N → I. Li *et al.*^5^ suggested that this may be due to the “stochastic nature” of mechanical unfolding. They suggested that since N → I unfolding is observed at higher unfolding forces than the subsequent I → U unfolding, the I → U unfolding may sometimes occur at a force that is too low to be seen in the AFM force-extension traces.^5^ That is, the unfolding is likely always to occur *via* this intermediate but it may not be observed, particularly when N unfolds at high forces. Thus if the simulations are correct we might expect to see fewer I → U transitions at lower pH.

To our surprise, the unfolding forces at all pH values are the same within error (Figure 3). This is true at all pulling speeds. The dependence of the unfolding force on the pulling speed remains unchanged, suggesting that there has been no change in the unfolding pathway. Note also that the proportion of I → U unfolding events remains about the same. Thus our results are in direct contradiction to the predictions from the steered molecular dynamics simulations.

AFM combined with protein engineering Ф-value analysis and molecular dynamics simulations has been previously used to solve the mechanical unfolding pathway of TNfn3, a homologous fnIII domain from the extracellular matrix protein tenascin.^13^ The results suggest that the unfolding of fnIII domains is a complicated multi-step process. The major barrier to a forced unfolding event in TNfn3 is the conformational transition from a twisted to an aligned state, which involves the breaking of several key hydrogen bonds along the peripheral strands (A-B strands and some between the G and F-strands). But there is also significant loss of hydrophobic side-chain packing interactions of residues in the A, B and G-strands, and importantly there is significant core re-packing. Thus, hydrophobic contacts of the buried residues and the hydrogen bonding interactions along peripheral strands are both apparently critical to the mechanical stability of TNfn3. TNfn3 and FNfn10 fold into essentially identical tertiary structures encompassing seven beta-strands running in two antiparallel beta sheets. A structural alignment of TNfn3 and FNfn10 reveals that these two fnIII domains have essentially the same hydrogen bonding patterns^14^ and molecular dynamics simulations have suggested that they have similar forced unfolding pathways. It is therefore reasonable to anticipate that these two fnIII domains will have the same molecular determinants for mechanical stability. We have made a “core-swap” version of FNfn10, with the core of Tnfn3 which is significantly more stable than Fnfn10 itself.^15^ Considering these together, it seems that the mechanical stability of both FNfn10 and TNfn3 is likely to be associated with the complex interplay between key peripheral hydrogen bonds and hydrophobic effects from the packing of buried residues.

Molecular dynamics simulations have previously proved to be of great value in predicting and understanding the behavior of proteins placed under mechanical stress.^6^^,^^7^^,^^11^^,^^13^^,^^16^^,^^17^ However, our results suggest that in the case of the simulations of forced unfolding of FNfn10 tested here,^6^ the relative strength and importance of electrostatic and hydrophobic components may not be adequately described. It is possible that this discrepancy lies in the different timescale of the simulations and the experiments. AFM experiments are typically performed at extension rates of ∼1 μm s^−1^, whereas the timescale of molecular dymamics simulations allows for full extension of the protein in 1 or 2 ns, equivalent to a pulling speed of several m s^−1^, many orders of magnitude faster. Many slow conformational changes in proteins will not be observed in simulations on the nanoseconds timescale. Furthermore, the pathway of forced unfolding may vary as a function of the loading rate.^18^^,^^19^ Our study serves to emphasize the point that simulations need constantly to be benchmarked against experiment. However, the fact that simulators are prepared to make *ab initio* predictions that can be tested experimentally can only serve to improve our understanding of molecular mechanisms underlying mechanical strength in proteins.

## Figures and Tables

**Figure 1:**
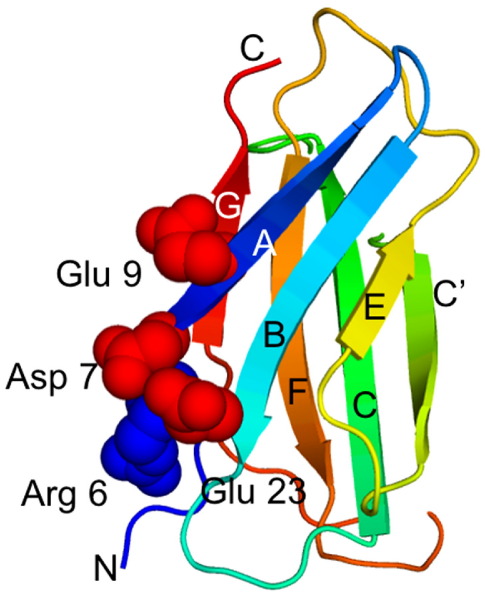
The structure of FNfn10. The strands are labeled and the N and C termini shown. The Asp and Glu residues in the A and B strands that are protonated at pH 4.5 are in red.

**Figure 2:**
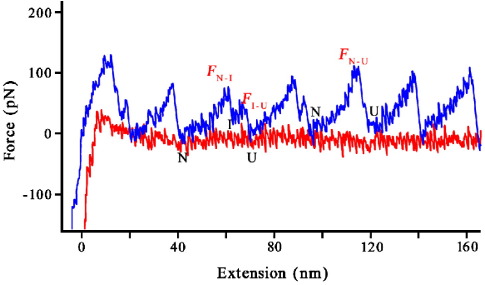
A typical forced unfolding trace of a FNfn10 polyprotein. Two kinds of unfolding events are observed. The FNfn10 module unfolds either by a two-state unfolding mechanism (N → U, unfolding force = *F*_N→U_) or a three-state unfolding mechanism *via* an intermediate (N → I → U, unfolding forces = *F*_N→I_ and *F*_I→U_), where N, I and U are the native, intermediate and unfolded states, respectively. All AFM unfolding experiments of FNfn10 were carried out using a polyprotein construct comprising eight identical FNfn10 domains (96 residues long, residues Val1416 to Ile1511), cloned and expressed by standard methods.^20^ Standard equilibrium denaturation experiments were undertaken to show that the FNfn10 domains were stable and folded in the polyprotein^21^ (data not shown). Fernandez and co-workers^5^ have previously investigated the unfolding pattern of wild-type and mutant forms of FNfn10 at pH 7.4 (PBS) but with a chimeric polyprotein construct consisting of the FNfn10 module and the I27 module (FNfn10-I27)_4_. The unfolding forces collected here are the same, within error as those observed in (FNfn10-I27)_4_.

**Figure 3:**
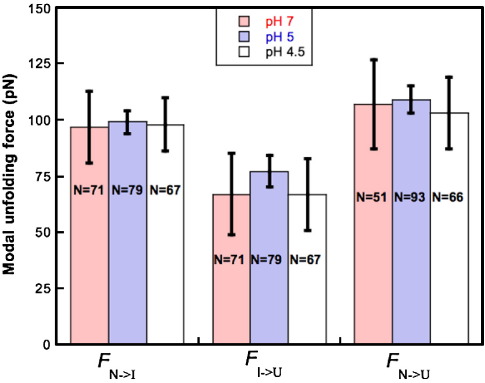
The unfolding forces of FNfn10 are independent of pH. The unfolding forces for the N → U transition (*F*_N→U_) the N → I transition (*F*_N→I_) and the I → U transition (*F*_I→U_) are shown at pH 7.0 (shaded red bars), 5.0 (shaded blue bars) and 4.5 (open bars). The number of unfolding events observed (*N*) is given. The error bars show ± 1 s.d. There is no significant difference in unfolding forces, or in the relative frequency of I → U unfolding events at any pH. All the data were collected at a single pulling speed (1000 nm s^−1^) using the same cantilever on the same day to eliminate errors in cantilever calibration. All force measurements were performed as described previously using a Molecular Force Probe-1D (Asylum Research, Santa Barbara, CA) and analysed by standard methods.^13^
